# The *ZtvelB* Gene Is Required for Vegetative Growth and Sporulation in the Wheat Pathogen *Zymoseptoria tritici*

**DOI:** 10.3389/fmicb.2019.02210

**Published:** 2019-10-01

**Authors:** Anna M. M. Tiley, Hannah J. White, Gary D. Foster, Andy M. Bailey

**Affiliations:** ^1^Molecular Plant Pathology and Fungal Biology, School of Biological Sciences, University of Bristol, Bristol, United Kingdom; ^2^School of Biology and Environmental Science, O’Brien Centre for Science, University College Dublin, Dublin, Ireland

**Keywords:** ascomycete, *Aspergillus nidulans*, *Zymoseptoria tritici*, Septoria Tritici Blotch, velvet, light

## Abstract

The ascomycete fungus *Zymoseptoria tritici* is the causal agent of Septoria Tritici Blotch (STB), a major disease of wheat across Europe. Current understanding of the genetic components and the environmental cues which influence development and pathogenicity of this fungus is limited. The *velvet B* gene, *velB*, has conserved roles in development, secondary metabolism, and pathogenicity across fungi. The function of this gene is best characterised in the model ascomycete fungus *Aspergillus nidulans*, where it is involved in co-ordinating the light response with downstream processes. There is limited knowledge of the role of light in *Z. tritici*, and of the molecular mechanisms underpinning the light response. We show that *Z. tritici* is able to detect light, and that the vegetative morphology of this fungus is influenced by light conditions. We also identify and characterise the *Z. tritici velB* gene, *ZtvelB*, by gene disruption. The Δ*ztvelB* deletion mutants were fixed in a filamentous growth pattern and are unable to form yeast-like vegetative cells. Their morphology was similar under light and dark conditions, showing an impairment in light-responsive growth. In addition, the Δ*ztvelB* mutants produced abnormal pycnidia that were impaired in macropycnidiospore production but could still produce viable infectious micropycnidiospores. Our results show that *ZtvelB* is required for yeast-like growth and asexual sporulation in *Z. tritici*, and we provide evidence for a role of *ZtvelB* in integrating light perception and developmental regulation in this important plant pathogenic fungus.

## Introduction

*Zymoseptoria tritici* (synonym *Mycosphaerella graminicola* or *Septoria tritici*) is an ascomycete fungus in the class Dothideomycetes, order Capnodiales, family Mycosphaerellaceae and genus *Zymoseptoria* (Quaedvlieg et al., 2011). This fungus is the causal agent of the foliar disease of wheat, Septoria Tritici Blotch (STB) (Eyal et al., 1987; Adams et al., 2010; Ponomarenko et al., 2011; Dean et al., 2012). *Z. tritici* is currently the greatest threat to European wheat production, and an estimated 70% of annual EU fungicide usage is targeted against it (Orton et al., 2011; Fones and Gurr, 2015; Torriani et al., 2015). Despite its economic importance, there are currently no fully effective ways to control *Z. tritici*, and all commercially available wheat varieties are susceptible to this pathogen to some degree (AHDB, 2018). Chemical application is the main method used to control *Z. tritici*, however, the pathogen is able to develop resistance to the fungicides currently used against it (Torriani et al., 2015). There is therefore an urgent need to develop novel strategies to control this major threat to European wheat production.

*Zymoseptoria tritici* spreads to susceptible host plants by asexual pycnidiospores and sexual ascospores which, following germination, infect the wheat leaf via the stomata (reviewed in Brennan et al., 2019). The lifestyle of the fungus is characterised by a symptomless latent period which typically lasts 10–14 days post-infection (dpi) (Eyal et al., 1987). This is followed by a switch to necrotrophic growth at 14–21 dpi, which presents as chlorotic and necrotic lesions on the wheat leaves. Dark brown asexual fruiting bodies (pycnidia) develop within these necrotic lesions at approximately 28 dpi, marking the onset of asexual sporulation (Eyal et al., 1987; Ponomarenko et al., 2011). The pycnidia produce two types of asexual spores (macropycnidiospores and micropycnidiospores) which are dispersed by rainsplash to neighbouring plants (Sprague, 1950; Eyal et al., 1987; Ponomarenko et al., 2011). Asexual sporulation is a key contributing factor behind the success of *Z. tritici* as a pathogen; it enables an increase of potential inoculum in the field and promotes the rapid spread of virulent strains within one growing season. Targeting this life cycle stage is therefore a promising strategy for future control methods.

Previous research focusing on *Z. tritici* development has shown that light is an environmental cue which may regulate key processes such as vegetative growth and asexual sporulation in this pathogen. For example, findings by Choi and Goodwin (2011) demonstrated that *Z. tritici* produces significantly more vegetative aerial hyphae under blue light conditions compared to red light conditions. In addition, aerial mycelial production was significantly reduced in dark conditions. Our previous findings have also highlighted the possible role of UV-A light in initiating asexual sporulation *in vitro* (Tiley et al., 2018). Therefore, light may be an environmental cue which is detected by this pathogen and used to influence developmental processes.

In other fungi, light has been shown to be an important environmental signal that regulates a range of developmental and metabolic processes (Idnurm et al., 2010; Rodriguez-Romero et al., 2010; Fuller et al., 2015). Fungi detect light through the use of specialised photoreceptor proteins that interact with downstream proteins to coordinate processes such as metabolism and development. One of the best studied model fungi is the ascomycete *Aspergillus nidulans*, which is in the class Eurotiomycetes, order Eurotiales, family Aspergillaceae and genus *Aspergillus*. In *A. nidulans*, the fungal photoreceptor proteins have been shown to be associated with *velvet* regulator proteins. In total, four *velvet* genes (*veA*, *velB*, *velC*, and *vosA*) have been identified in this fungus, and all share a characteristic *velvet* DNA binding motif (Mooney and Yager, 1990; Ni and Yu, 2007; Park et al., 2012).

In *Aspergillus nidulans*, the *velvet* proteins interact with each other and non-*velvet* proteins to form homodimer, heterodimer and trimeric complexes which regulate metabolism, and development. For example, the VelB protein can form a VelB/VosA complex that negatively regulates asexual spore maturation via the sporulation genes *brlA* and *wetA* (Ni and Yu, 2007; Bayram et al., 2008; Park et al., 2012). The VelB protein also forms a VelB/VeA/LaeA trimeric complex that co-ordinates the light response in *A. nidulans* via downstream developmental and secondary metabolism pathways (Bayram et al., 2008).

The velvet family of regulatory proteins are unique to fungi and homologues have been identified in many other ascomycete and basidiomycete species (Ni and Yu, 2007). These proteins have been shown to have roles in vegetative differentiation, pathogenicity, and secondary metabolism in species including *Curvularia lunata*, *Fusarium graminearum*, *Botrytis cinerea* and *Magnaporthe oryzae* (Jiang et al., 2012; Yang et al., 2013; Kim et al., 2014; Gao et al., 2017). This therefore suggests a conserved role for the velvet family in development and secondary metabolism among fungi.

To date, the only *velvet* gene characterised in *Z. tritici* is the *veA* homologue, *MVE1*. Mutants with deletions of *MVE1* have defects in melanin biosynthesis, aerial hyphal growth, and hydrophobicity. In addition, Δ*mve1* mutants do not respond to light cues for the development of aerial mycelia (Choi and Goodwin, 2011). This indicates that the *Z. tritici MVE1* gene has similar roles to the *A. nidulans veA* gene in the coordination of light perception with downstream developmental processes.

Here we identify and characterise the *velvet B-*like gene in *Z. tritici*, *ZtvelB.* We show that *Z. tritici* has a complete set of *velvet*-like homologues (*MVE1*, *ZtvelB*, *ZtvelC*, and *ZtvosA*). We investigate the role of *ZtvelB* in this pathogen by constructing Δ*ztvelB* knock-out mutants. Our results suggest that *ZtvelB* has roles in vegetative growth and asexual sporulation in *Z. tritici*. These findings provide further evidence for the importance of *velvet*-like genes in *Z. tritici*, linking the perception of light to the production of asexual pycnidiospores, and we provide new avenues to explore novel control methods against this pathogen.

## Materials and Methods

### Identification of *veA*, *velB*, *velC*, and *vosA* Genes in *Z. tritici*

The published genome of *Z. tritici* IPO323 was used throughout this study to identify and characterise potential *velvet* gene homologues in this pathogen (Goodwin et al., 2011). The *A. nidulans velvet* proteins VeA, VelB, VelC, and VosA were queried against the *Z. tritici* genome database^1^ using the tblastn and Filtered Models (transcripts) algorithms. More than one BLAST match occurred in *Z. tritici* for each of the four *A. nidulans velvet* protein sequences. In order to identify the true *velvet* gene homologues in *Z. tritici*, each *A. nidulans velvet* gene, along with homologues from *M. oryzae* and *B. cinerea*, and the matches in *Z. tritici* were aligned using Clustal X version 2.0 (Larkin et al., 2007). Molecular evolutionary genetics (MEGA) 6 software was used to create a neighbour-joining phylogenetic tree (Tamura et al., 2013). The *Z. tritici velvet* protein sequences were also analysed for presence of the *velvet* superfamily factor domain (pfam11754).

### Construction of *ZtvelB* Knock-Out Plasmids

A knock-out vector for targeted deletion of *ZtvelB* (pΔ*ztvelB*) was constructed using yeast-based homologous recombination. The primers used to generate pΔ*ztvelB* are listed in Supplementary Table S2 and Supplementary Figure S1. The pΔ*ztvelB* vector consisted of a pCAMBIA0380_YA (yeast-adapted) backbone, with the Hygromycin-*trpC* resistance cassette from pCB1003 (Carroll et al., 1994) flanked by two 1.5 kb regions targeting the *ZtvelB* locus. The flanking regions and Hygromycin-*trpC* resistance cassette were amplified using Phusion^®^ High-Fidelity DNA Polymerase (Thermo Fisher Scientific).

A Zymoprep^TM^ Yeast Plasmid Miniprep II kit (Zymo Research) was used to recover plasmid DNA from *Saccharomyces cerevisiae*, rescued into *Escherichia coli ccdB* or DH5α cells and isolated using the Gene JET Plasmid Miniprep Kit (Thermo Fisher Scientific) or Gene JET Plasmid Midiprep Kit (Thermo Fisher Scientific) following the manufacturer’s instructions. Correct plasmid assembly was initially confirmed by PCR and further confirmed by sequencing, using the primers detailed in Supplementary Table S2 and Supplementary Figure S1.

### *Agrobacterium-*Mediated Transformation

The pΔ*ztvelB* knock-out vector was transformed into *Agrobacterium tumefaciens* LBA1126 and AGL1 cells. *Agrobacterium*–mediated transformation was then used to transform the *Z. tritici* IPO323 strain following the protocol outlined in Derbyshire et al. (2015).

### Confirmation of Δ*ZtvelB* Mutants

Primary screening of the Δ*ztvelB* knock-out mutants was carried out by growing the strains on YPDA agar (10 g/L yeast extract, 20 g/L peptone, 20 g/L glucose, and 20 g/L technical agar) supplemented with Hygromycin B (100 μg/ml) and Timentin^TM^ (100 μg/ml). Potential mutants were then sub-cultured at least three times to single colonies. Successful deletion of the *ZtvelB* gene was confirmed by PCR (Supplementary Figure S2). Wild-type and knock-out mutant fungal DNA was extracted using the protocol outlined in Liu et al. (2000). Double PCR was carried out using two primer pairs; the first primer pair was designed to amplify the wild-type *ZtvelB* gene, and the second pair to amplify the successful replacement of *ZtvelB* with the Hygromycin-*trpC* resistance cassette. Primers used for knock-out confirmation are detailed in Supplementary Table S2 and Supplementary Figure S1.

### *In vitro* Experiments

*Z. tritici* strains were cultures on either PDA (24 g/L potato dextrose broth and 20 g/L technical agar), Czapek Dox-V8 juice (CDV8) agar (46 g/L Czapek Dox agar, 200 ml/L V8^®^ Original vegetable juice (Campbell’s), 3 g/L calcium carbonate and 10 g/L technical agar), or YPDA agar. Cultures were incubated under either a light: dark cycle (LD) (white light supplemented with UV-A light, 12:12 photocycle) or darkness (DD) (conditions as above but plates wrapped in foil) at 20°C.

Sporulation experiments were carried out on wheat extract agar (WEA) (37.5 g/L homogenised 21 day-old wheat leaves cv. Riband, 20 g/L technical agar) under 12:12 LD cycles at 20°C for up to 70 days as described previously in Tiley et al. (2018).

Liquid cultures were carried out by inoculating a 250 ml conical flask containing 50 ml PDB with a 7.5 mm diameter agar plug of *Z. tritici* excised from a 10-day-old CDV8 plate. This time point was chosen as it is when the IPO323 strain displays a hyphal morphology which is most similar to the Δ*ztvelB* mutants. The cultures were incubated at 20°C and 200 rpm for up to 10 days.

### *In planta* Experiments

Virulence of the *Z. tritici* strains was compared using attached wheat leaf inoculations, as described in Keon et al. (2007) and Tiley et al. (2018). The susceptible wheat cultivar Riband was used for all experiments. As the Δ*ztvelB* mutants did not display yeast-like growth, it was necessary to modify the inoculation method. Wheat leaves were directly infected with a 7.5 mm agar plug of the *Z. tritici* IPO323 strain or Δ*ztvelB* mutants taken from a 10-day-old CDV8 plate, and dipped in 500 μl filter-sterilised 0.1% Tween 20 to facilitate spreading of the fungal hyphae onto the leaf (Motteram et al., 2009). A plug from a CDV8 plate with the IPO323 strain was used as a positive control, and a clean plug with no fungal growth was used as a negative control. Each plug was swabbed fungal side down over the leaf surface ten times. Infected plants were sealed inside a clear 40 μm thick autoclave bag and kept in high humidity for 72 h at 20°C under a 16:8 light:dark cycle.

Comparison of virulence of the *Z. tritici*Δ*ztvelB* mutants and IPO323 strain was assessed by monitoring and recording disease progression every 2–3 days. Disease symptoms on the infected leaves were scored from 1 to 5 using a modified version of the scale outlined in Skinner (2001). Infected wheat leaves were harvested at 28 days post infection to compare pycnidia and pycnidiospore production as described previously in Tiley et al. (2018). This procedure was used for 7–12 leaf sections per strain of *Z. tritici* and each leaf was treated as a technical replicate.

For micropycnidospore growth assays, between 3 and 5 infected leaves were pooled for each strain and vortexed in 1 ml sterile deionised water (SDW). A 200 μl sample from each cell suspension was spread on PDA supplemented with Hygromycin B (100 μg/ml) and Timentin^TM^ (100 μg/ml). The petri dishes were incubated in DD at 20°C for 16–20 days.

### Microscopy and Photography

*Z. tritici* spores and vegetative hyphae were stained with lactophenol cotton blue for analysis using light microscopy. Five microlitre of the stain was put on a glass microscope slide followed by 5 μl of the fungal cell suspension and a cover slip. The specimen was left to stand for 1 min before microscope analysis.

Samples were observed using a Leica DM5500 B microscope or Leica M205 FA stereo microscope connected to a Leica DFC310 FX digital camera, and captured using Leica Application Suite software V 4.4. Images of leaf samples were taken using a Canon EOS 100D camera with EF-S 18–55 mm f/3.5–5.6 IS STM Lens.

### Statistical Analyses

Comparison of pycnidia numbers was carried out using IBM SPSS Statistics 24 (IBM Corp., 2016). A linear mixed effects model with mean pycnidia number as the dependent variable was used with fungal strain as the independent variable, and biological replicate included as a random effect to account for variation between replicates. The assumption of homogeneity of variance was assessed using Levene’s, Brown – Forsye and Welch ***F*** tests. Tukey’s honest significant difference (HSD) test at the 5% significance level was used for ***post hoc*** comparison between strains to account for multiple comparisons. Count data collected from micropycnidospore analyses was highly zero-inflated. We used a zero-inflated mixed effects model with a negative binomial error structure, to deal with overdispersion, and a log link function. Biological replicate was included as a random effect. This model was fitted in R v3.5.1 (R Core Team, 2018) using the ***glmmTMB*** function in the R package ‘***glmmTMB***’ (Brooks et al., 2017). Tukey’s HSD (HSD) ***post hoc*** test at the 5% significance level was used to compare micropycnidospore**s** production between strains.

## Results

### The Role of Light for Vegetative Growth in *Z. tritici*

In order to investigate the role of light in *Z. tritici* development, vegetative growth was analysed on PDA, YPDA, and CDV8 agar under two different light regimes; 12:12 light:dark (LD) and darkness (DD). The cultures were kept under each light regime at 20°C for 10 days, and experiments were performed in triplicate.

**FIGURE 1:**
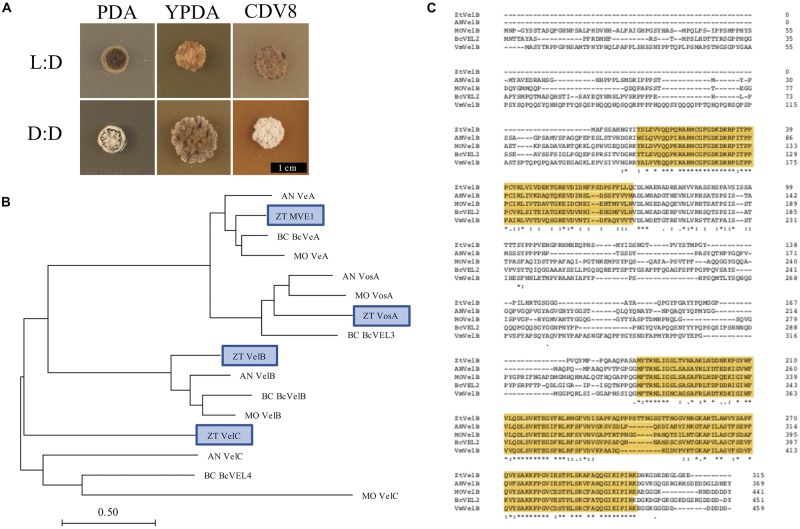
**(A)** *Zymoseptoria tritici* IPO323 vegetative growth on PDA, YPDA, and CDV8 agar at 20°C, under LD or DD conditions, at 10 days post-inoculation (dpi). Cultures grown under LD develop as yeast-like cells which start to melanise after 7–10 dpi Under DD conditions, the *Z. tritici* cultures melanise and form grey and white aerial hyphae. **(B)** Neighbour-joining phylogenetic trees of *A. nidulans velvet* proteins and their relatedness to potential *Z. tritici* homologues. Bar represents 0.5 amino acid substitutions per site. Blue boxes indicate the candidate orthologues identified in *Z. tritici.* The previously described *Z. tritici* MVE-1 protein (Choi and Goodwin, 2011) was identified as a close relative to the *A. nidulans* VeA protein. *Z. tritici* protein ID numbers 103692, 86705, and 108742 were identified as potential homologues for the *A. nidulans* VelC, VelB, and VosA proteins, respectively. **(C)** Clustal Omega alignment of *velB* protein sequences from *Z. tritici* (ZT), *A. nidulans* (AN), *M. oryzae* (MO), *B. cinerea* (Bc), and *V. mali* (Vm). The characteristic velvet superfamily domains are highlighted in yellow, asterisks identify identical residues, colons identify conserved residues and periods identify semi-conserved residues.

Fungal colonies incubated under LD grew as yeast-like cells from 0 to 10 dpi. Between 7 and 10 dpi, the fungal colonies began to melanise, and develop grey and white aerial hyphae. In contrast, cultures grown under DD only remained as yeast-like cells for approximately 5–7 days. By 10 dpi, all dark-grown colonies had begun to melanise and form white or grey aerial hyphae (Figure 1). The most striking phenotypic difference was between IPO323 cultures grown on CDV8 agar and PDA under LD or DD. Under LD conditions, the IPO323 colonies grown in light on CDV8 agar and PDA remained yeast-like with some melanisation at 10 dpi. However, cultures incubated under DD conditions on these two media types were no longer yeast-like, and instead produced abundant grey or white aerial hyphae from a melanised base.

### Identification of VELVET Protein Homologues in *Z. tritici*

Homologues to the four *A. nidulans velvet*-encoding genes were successfully identified in *Z. tritici* using the *A. nidulans veA*, *velB*, *velC*, and *vosA* FASTA protein sequences. Each of the blast searches against the *Z. tritici* IPO323 genome (Goodwin et al., 2011) consistently gave a combination of only the protein ID numbers 37276, 86705, 103692, or 108742 (Supplementary Table S1).

As more than one potential homologue was identified in *Z. tritici* for each *A. nidulans velvet* gene, phylogenetic trees were generated to infer relationships. Additional protein sequences from known fungal *velvet* genes documented in the literature were included in the analysis in order to increase certainty of identification (Figure 1). This analysis assigned a single hit in *Z. tritici* to *veA*, *velB*, *velC*, and *vosA* (Table 1) and also supports the previous identification of protein ID number 37276 (*MVE-1*) as the *Z. tritici* homologue to the *A. nidulans veA* gene (Choi and Goodwin, 2011). An NCBI conserved domain search (CD-search) for the four *Z. tritici velvet* candidates showed that all four of the *Z. tritici* proteins contain the velvet factor (pfam11754), which is a key component of all *velvet* genes. Further analysis of the *Z. tritici velB* gene (*ZtvelB*) amino acid sequence also revealed a high similarity to known *velB* proteins from *A. nidulans*, *M. oryzae*, *B. cinerea*, and *Valsa mali* (Figure 1).

**TABLE 1:** The four *velvet* genes chosen for knock-out in *Zymoseptoria tritici*, listing each gene name, the protein ID number, transcript size, chromosome location, and protein information as annotated in the *Z. tritici* genome database (Goodwin et al., 2011).

**Gene name**	**Protein ID number**	**Transcript size (bp)**	**Chromosome location**	**Protein information**
*ZtveA* (*MVE-1*)	37276	810	chr_2: 2995014-2995823	N/A
*ZtvelB*	86705	948	chr_7:190195-191200	N/A
*ZtvelC*	103692	1597	chr_3:1523180-1524776	Serine/threonine protein kinase
*ZtvosA*	108742	1939	chr_3:2480800-2482921	Signal transducing adaptor protein STAM/STAM2

*The *ZtveA* gene was identified as *MVE-1* (Choi and Goodwin, 2011).*

### Generation of *Z. tritici ΔztvelB* Mutants

In order to investigate the role of the *Z. tritici velvet* genes identified, the *ZtvelB* knock-out vector (pΔ*ztvelB*) was constructed in a yeast-adapted version of pCAMBIA_0380 by yeast-based homologous recombination, and transformants of IPO323 were generated using *Agrobacterium-*mediated transformation. Two distinct colony morphologies were apparent on the transformation plates, with one form being the typical yeast-like growth, and the other being purely filamentous. Colonies displaying each type of morphology were investigated.

The Δ*ztvelB* transformants were purified by subculture on selective media and confirmed by double PCR using two different primer pairs (Supplementary Figures S1, S2 and Supplementary Table S2). Successful disruption was indicated by both loss of the wild-type *ZtvelB* amplicon and gain of the knockout amplicon (Supplementary Figures S1, S2). This showed that the colonies with filamentous morphology were the Δ*ztvelB* mutants. The Δ*ztvelB* mutants were successfully generated in the wild-type IPO323 background with 10–20% targeting efficiency. Throughout the study, three independent deletion strains were analysed against the parental IPO323 strain.

### Yeast-Like Vegetative Growth Is Disrupted in the Δ*ztvelB* Mutants

**FIGURE 2:**
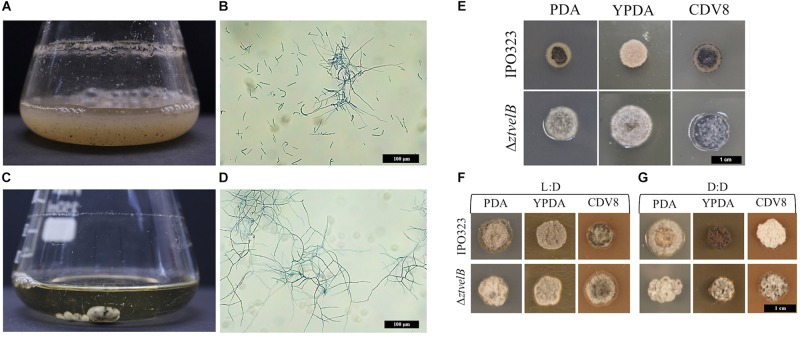
Comparison of vegetative growth of the *Z. tritici* IPO323 and the Δ*ztvelB* mutant strains. Images representative of three independent Δ*ztvelB* strains tested across three independent experiments. **(A–D)** *Z. tritici* IPO323 strain and Δ*ztvelB* mutant growth in PDB at 20°C on a rotary shaker at 200 rpm, 7 days post-inoculation (dpi) stained with lactophenol cotton blue. **(A,B)** The IPO323 strain forms a cloudy suspension composed of short yeast-like cells and branched hyphal cells. **(C,D)** The Δ*ztvelB* mutants form mycelial clumps composed of branched hyphal cells and no yeast-like cells. **(E)** IPO323 and Δ*ztvelB* mutant growth on solid media at 20°C under LD conditions, at 7 dpi. The IPO323 strain grows as yeast-like cells which melanise on PDA and CDV8. The Δ*ztvelB* mutants only grow as grey and white aerial hyphae from a melanised base. **(F)** IPO323 and Δ*ztvelB* mutant growth under LD or DD conditions at 10 dpi. Under LD, the IPO323 colonies melanise and produce grey and white aerial hyphae by 10 dpi. **(G)** Under DD the IPO323 strain melanises and forms grey aerial hyphae on PDA. On YPDA, DD colonies produce fewer aerial hyphae than under LD. On CDV8, DD colonies produce more white aerial hyphae than under LD. In contrast, the Δ*ztvelB* mutant only grow as grey and white aerial hyphae which are phenotypically similar under both LD and DD conditions.

The role of the *ZtvelB* gene in development was assessed by comparing vegetative growth of the Δ*ztvelB* mutants to the parental IPO323 strain. In liquid PDB, the IPO323 strain formed a pale pink cloudy suspension which melanised after 5–7 dpi. The IPO323 cells initially grew as yeast-like cells which later formed branching hyphae. The Δ*ztvelB* mutants did not grow as yeast-like cells in liquid media, and instead formed a light-coloured mycelial mass which melanised to form a dark green-to-black turbid culture. Microscopy of the Δ*ztvelB* cultures showed that the mutant strains did not produce yeast-like cells and were instead comprised of branching hyphae (Figure 2).

Solid media experiments were carried out by inoculating the agar with a 2.5 μl cell suspension taken from a 7 to 10 day-old Δ*ztvelB* or IPO323 liquid culture. YPDA and CDV8 media were specifically chosen for *in vitro* tests as these promote *Z. tritici* yeast-like growth *in vitro*, and PDA was selected as it induces hyphal growth. The mutants were grown under 12:12 LD or DD conditions to assess the impact of deleting *ZtvelB* on light detection in *Z. tritici*.

On all three solid media types tested under LD conditions, the IPO323 strain initially grew as colonies composed of pale pink vegetative yeast-like cells (Figure 2). These IPO323 colonies melanised and produced grey and white aerial hyphae by 10 dpi. Under DD conditions, the IPO323 strain also began as colonies composed of pale pink-coloured yeast-like cells which melanised and produced aerial hyphae by 10 dpi. On YPDA the IPO323 phenotype produced fewer aerial hyphae under DD compared to LD, and on CDV8 the IPO323 strain produced visibly more white aerial hyphae under DD than LD. The Δ*ztvelB* mutant strains did not form yeast-like cells on any of the three solid media tested under either LD or DD conditions. Instead, the Δ*ztvelB* mutants grew as short, white and grey aerial hyphae from a black melanised base (Figure 2). Growth under LD or DD conditions also did not affect morphology of the Δ*ztvelB* mutants, and these strains were phenotypically similar under both of the light conditions tested (Figure 2).

### *ΔztvelB* Mutants Have Defects in Asexual Sporulation *in vitro*

In order to assess the role of *ZtvelB* in asexual sporulation in *Z. tritici*, the IPO323 and Δ*ztvelB* mutant strains were grown on WEA under 12:12 LD conditions. WEA was selected as it enables the uncoupling of asexual sporulation from the ability to cause infection.

Under LD conditions, the IPO323 strain developed pycnidia similar to those observed *in planta* and previously documented *in vitro* (Tiley et al., 2018). Pycnidia were visible on the IPO323 petri dishes at 28 days post-inoculation, but the cultures were incubated for 69 days in order to promote further pycnidia development and cirrhus release. The centre of the IPO323 colonies initially grew as yeast-like cells that by 21 dpi had developed as white aerial hyphae extending from a melanised base. Hyaline cirrhus similar to that documented for *Z. tritici in planta* experiments was observed as exuding from the centre of these colonies by 69 dpi.

**FIGURE 3:**
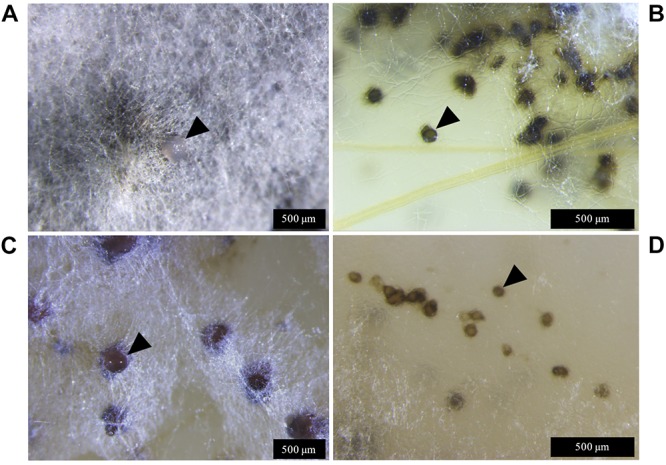
*Zymoseptoria tritici* IPO323 and the Δ*ztvelB* mutant pycnidia on wheat extract agar (WEA) at 20°C under LD conditions at 69 days post-inoculation. Images representative of three independent Δ*ztvelB* strains tested across two experiments. **(A)** The IPO323 strain forms white aerial hyphae from a melanised based from which hyaline cirrhus is produced (indicated by arrowheads). **(B)** Develops spherical black/brown pycnidia beneath the surface of the agar (indicated by arrowheads). **(C)** The Δ*ztvelB* mutants form white aerial hyphae with large abnormal pycnidia-like structures on the surface of the agar. The pycnidia-like structures exude clear droplets (indicated by arrowheads). **(D)** The Δ*ztvelB* mutants also form pycnidia-like structures similar to IPO323 beneath the surface of the agar (indicated by arrowheads).

Lateral hyphae radiated away from the centre of inoculation and formed white hyphal knots by 21 dpi. These hyphal knots developed into dark brown globose pycnidia by 28 dpi, which were mostly embedded below the surface of the agar (Figure 3).

The Δ*ztvelB* mutants were all unable to form yeast-like cells on the WEA when incubated under LD conditions. Instead, these mutants formed a thick mat of white hyphae over the surface of the agar. The Δ*ztvelB* mutants formed hyphal knots on the agar and some of these produced pycnidia-like structures superficially similar to IPO323 (Figure 3). However, the Δ*ztvelB* structures were mostly embedded within the thick mat of hyphae formed by the fungus and were not spherical in shape. The mutant pycnidia secreted a clear liquid, but this was unlike the hyaline cirrhus produced by the IPO323 strain (Figure 3).

In addition to the abnormal pycnidia-like structures located within the hyphal mat, the Δ*ztvelB* mutants also formed pycnidia phenotypically similar to the IPO323 strain along the colony edge. These pycnidia were located below the surface of the agar and were similar in appearance to the IPO323 pycnidia. Due to the location of these structures within the agar, it was not possible to excise the Δ*ztvelB* pycnidia for further analysis.

### The Δ*ztvelB* Mutants Are Able to Cause Disease *in planta*

In order to assess the role of *ZtvelB* in virulence, susceptible wheat plants (cv. Riband) were infected with the IPO323 strain or the Δ*ztvelB* mutants, and disease outcome was measured. Between seven and twelve wheat plants were infected for each strain and experiments were repeated in triplicate.

**FIGURE 4:**
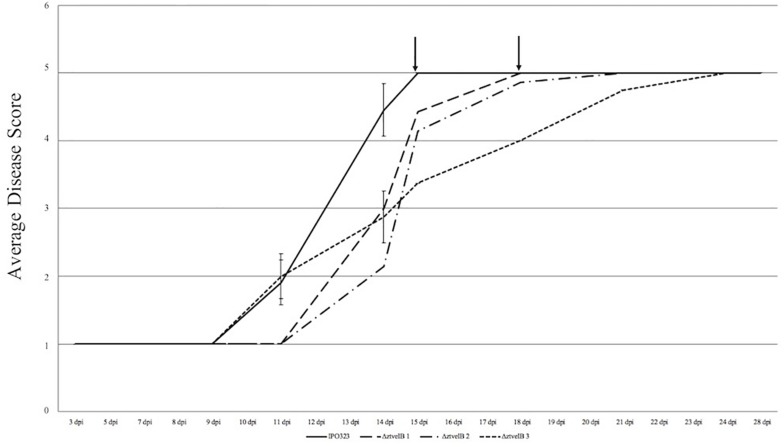
Average timing of *Z. tritici* infection of the IPO323 strain compared to the Δ*ztvelB* mutants at 0–28 dpi. Three independent Δ*ztvelB* strains (dotted lines) were tested against the parental IPO323 strain (straight line), and experiments were repeated in triplicate with 7–12 technical repeats each time. Graph representative of three biological replicates. Wheat leaves (cv. Riband) were inoculated with a plug extracted from a 10-day-old CDV8 agar plate dipped in 0.1% Tween 20. Symptoms were scored on a scale of 1–5 ever 2–3 days. Bars represent standard error and black arrows indicate the point at which the IPO323 strain and first Δ*ztvelB* strain reached the fifth and final stage of infection. In all three experiments, the IPO323 strain produced symptoms before all three Δ*ztvelB* strains.

**FIGURE 5:**
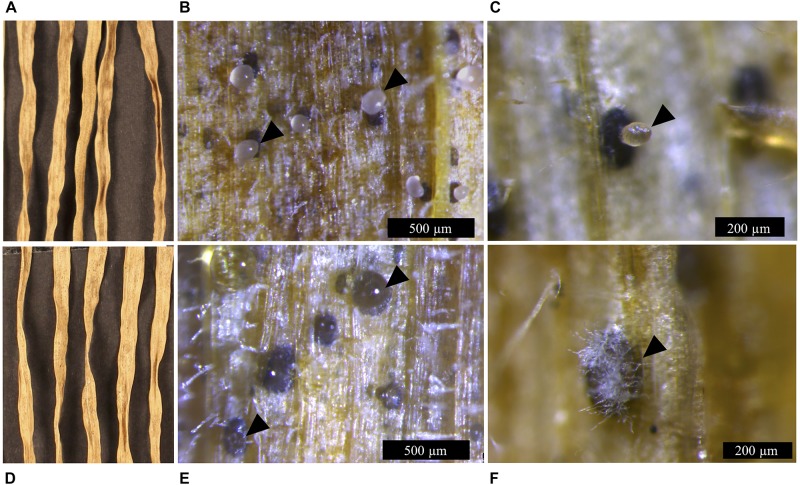
Infection symptoms of wheat leaves (cv. Riband) infected with the *Z. tritici* IPO323 strain and the Δ*ztvelB* mutants at 28 dpi. Images representative of three independent Δ*ztvelB* mutants tested against IPO323. Experiments were carried out in triplicate with 7–12 technical repeats each time. Leaves were inoculated with a plug extracted from a 10-day-old CDV8 agar plate dipped in 0.1% Tween 20. Samples were collected at 28 dpi and incubated under high humidity to induce cirrhus release. **(A)** IPO323 produces necrotic lesions after 28 dpi. **(B,C)** dark brown pycnidia on the infected wheat leaves which produce hyaline cirrhus from the ostiole (indicated by arrowheads). **(D)** the Δ*ztvelB* mutants produce necrotic lesions on wheat leaves. **(E,F)** pycnidia are dark brown in colour with clear cirrhus and some have white aerial hyphae on the exterior, developing from the ostiole region (indicated by arrowheads).

Disease progression was used to compare virulence between the IPO323 strain and the Δ*ztvelB* mutants. The first symptoms of disease, visible as chlorotic flecks, on leaves infected with IPO323 appeared at 3–11 dpi. Within 2–3 days of appearance, the chlorotic flecks coalesced to form larger chlorotic lesions at approximately 5–12 dpi. The chlorotic lesions developed into full necrotic lesions by 15 dpi which contained pycnidia (Figure 4 and Supplementary Figures S3, S5). The Δ*ztvelB* mutant strains were also able to produce the full sequence of disease (Figure 4 and Supplementary Figure S3). The leaves infected with the Δ*ztvelB* mutants all developed chlorotic and necrotic lesions, followed by pycnidia production (Figure 5). The timing of symptom appearance, however, was delayed (typically by 3–4 days) compared to IPO323. In all three biological replicates, the wild-type reached the fifth, and final stage of infection before all of the three Δ*ztvelB* mutant strains (Figure 4 and Supplementary Figure S3).

**FIGURE 6:**
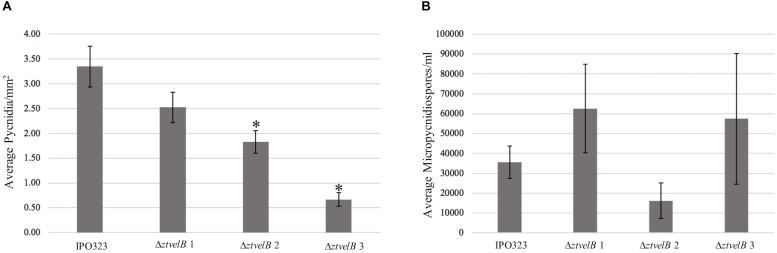
Comparison of the average pycnidia/mm^2^ and micropycnidiospores/ml produced by IPO323 compared to the three Δ*ztvelB* mutant strains at 28 dpi. Experiments were carried out in triplicate with 7–12 technical replicates each time. Bars represent standard error and an asterisk (^∗^) signifies strains significantly different to the IPO323 strain. **(A)** The Δ*ztvelB* 2 and Δ*ztvelB* 3 mutants produced significantly fewer pycnidia than the IPO323 strain at the 5% level. **(B)** The three Δ*ztvelB* mutants do not produce statistically significantly numbers of micropycnidiospores compared to the IPO323 strain at the 5% level.

As a second measure of virulence, the average number of pycnidia produced per mm^2^ (pycnidia/mm^2^) by IPO323 and the Δ*ztvelB* mutant strains was compared. There was no significant effect of biological replicate on pycnidia numbers between the strains (*n* = 108, *p* < 0.001). Reduction in the number of pycnidia produced by the three Δ*ztvelB* mutants compared to the IPO323 strain across all three biological replicates was not statistically significant at the 5% level. There was a significant difference between the average pycnidia/mm^2^ produced by the IPO323 strain (*n* = 30) and Δ*ztvelB* 2 (*n* = 27, standard error (SE) = 0.36, df = 104, *p* = 0.005), and Δ*ztvelB* 3 (*n* = 26, SE = 0.37, df = 104, *p* = < 0.001). However, there was no significant difference between the average pycnidia/mm^2^ produced by the IPO323 strain (*n* = 30) and the Δ*ztvelB* 1 mutant (*n* = 25, SE = 0.37, df = 104, *p* = 0.5; Figure 6).

### Δ*ztvelB* Mutants Produce Abnormal Pycnidia *in planta*

To assess whether the abnormal pycnidia-like structure results observed *in vitro* were consistent *in planta*, microscopy analyses were used to assess the Δ*ztvelB* pycnidia formed on the wheat leaves. Wheat leaves infected with the IPO323 strain contained dark brown spherical pycnidia which protruded from the sub-stomatal cavity (Figure 5). These structures contained a single ostiole from which cloudy cirrhus was released. On initial inspection, the Δ*ztvelB* mutants also produced pycnidia-like structures. These structures were dark brown in colour and they were located in the leaf substomatal cavity (Figure 5), however, close inspection showed that the Δ*ztvelB* pycnidia-like structures were developmentally abnormal compared to the IPO323 pycnidia.

The Δ*ztvelB* mutant pycnidia-like structures were not spherical in shape but instead appeared collapsed and sunken beneath the leaf surface. Some of the pycnidia had white aerial hyphae on the surface and protruding from the region of the ostiole opening (Figure 5). This hyphal pycnidia phenotype was only observed in the Δ*ztvelB* mutants and not in the pycnidia produced by the IPO323 strain. Closer observations of the infected leaves showed that in these instances, neither the leaf nor the mutant pycnidia were covered by a mat of fungal hyphae. This hyphal pycnidia morphology, therefore, could not be attributed to superficial fungal growth covering the pycnidia. Instead, our observations suggest that this phenotype was produced from the pycnidia itself.

The Δ*ztvelB* pycnidia secreted clear liquid droplets (hyaline cirrhus) at the opening of the ostiole, in contrast to the cloudy viscous cirrhus secreted by IPO323, similar to that observed in the *in vitro* analyses (Figure 5). Taken together, the results from the *in planta and in vitro* asexual sporulation experiments suggest that the *ZtvelB* gene may be required for normal pycnidia development.

### Macropycnidiospore Production Is Severely Disrupted in the Δ*ztvelB* Mutants

Previous descriptions of the asexual spores produced by *Z. tritici* characterise two spore types; large macropycnidiospores (35–98 μm × 1–3 μm in size, with 3–5 septa) and small micropycnidiospores (8–10.5 μm × 0.81 μm in size without septa) (Sprague, 1950; Eyal et al., 1987). Given that both the wild-type IPO323 strain and Δ*ztvelB* mutants produced cirrhus *in planta*, the production of macropycnidiospores and micropycnidiospores of the Δ*ztvelB* mutants was compared to IPO323.

**FIGURE 7:**
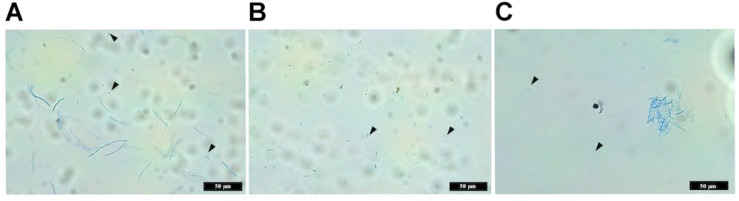
Images comparing macropycnidospores and micropycnidospores obtained from wheat leaves (cv. Riband) infected with the IPO323 strain and Δ*ztvelB* mutants. Spores were harvested from infected wheat leaves at 28 days post-inoculation and stained with lactophenol cotton blue. Images representative of all three Δ*ztvelB* knock-out mutant strains tested against the IPO323 strain. Experiments were carried out in triplicate with 7–12 technical replicates each time. Arrowheads indicate micropycnidiospores **(A)** IPO323 produces both macropycnidiospores and micropycnidiospores. **(B)** Δ*ztvelB* mutants only produce micropycnidospores. **(C)** Aggregation of micropycnidiospores obtained from the Δ*ztvelB* mutants.

The wild-type IPO323 produced a mixture of both macropycnidiospores (curved, septate cells, approximately 50 μm in length) and micropycnidiospores (short cells with no septa and less than 10 μm in length) (Figure 7). In contrast, no macropycnidiospores were recorded for any of the three Δ*ztvelB* mutant strains tested during spore measurements. Close observation of the cell suspension obtained from the infected leaves showed that, despite being severely disrupted in macropycnidiospores production, all three mutant strains still produced cells resembling micropycnidiospores. These spores were indistinguishable from the IPO323 strain micropycnidiospores in that they had no septa and measured less than 10 μm in length (Figure 7).

As deletion of Δ*ztvelB* disrupts macropycnidiospore production, the numbers of micropycnidiospores produced by the IPO323 strain and the three Δ*ztvelB* mutants was also compared. The IPO323 strain had an estimated count of 55270.8 micropycnidiospores (*n* = 31, SE = 1.27, df = 106). The Δ*ztvelB* 1 strain had an estimated count of 81633.91 micropycnidiospores (*n* = 26, SE = 1.27, df = 106), Δ*ztvelB* 2 of 48533.04 micropycnidiospores *n* = 27, SE = 1.43, df = 106), and Δ*ztvelB* 3 of 91126.14 micropycnidiospores *n* = 27, SE = 1.30, df = 106). There was no significant difference between the estimated means of the IPO323 strain with Δ*ztvelB* 1 (df = 104 *p* = 0.66), or Δ*ztvelB* 2 (df = 104 *p* = 0.99) or Δ*ztvelB* 3 (df = 104 *p* = 0.48).

### Micropycnidiospores Produced by the Δ*ztvelB* Mutants Can Cause Infection *in planta*

To verify that the cells obtained from the Δ*ztvelB* mutants were indeed micropycnidiospores, the cell suspensions were cultured *in vitro.* Each of the cell suspensions obtained from the three Δ*ztvelB* mutant strains produced fungal colonies with the Δ*ztvelB* mutant phenotype. The colonies were hyphal and melanised in appearance with white aerial hyphae, and no yeast-like growth (Supplementary Figure S4). This shows that the Δ*ztvelB* mutant micropycnidiospores are viable and capable of generating normal hyphal growth.

Virulence of the micropycnidiospores produced by the IPO323 strain and Δ*ztvelB* mutants was tested by infecting wheat leaves with the spore suspensions obtained from the infected leaves. The pooled suspensions were used to infect the leaves using a cotton bud. Between 2 and 3 leaves were infected per strain, and the experiment was carried out twice.

The spore suspensions obtained from the IPO323 strain and the three Δ*ztvelB* mutant strains tested all produced normal disease symptoms on the wheat leaves by 28 dpi (Supplementary Figure S5). The symptoms caused by the IPO323 spore suspensions caused chlorotic lesions which developed into necrotic lesions containing pycnidia. The micropycnidiospore suspensions obtained from the Δ*ztvelB* mutants were also able to cause chlorotic and necrotic lesions and produced the abnormal pycnidia phenotype observed previously *in planta* (Supplementary Figure S5).

Microscopy showed that the pycnidia formed on leaves infected with IPO323 secreted hyaline cirrhus, however, the pycnidia produced by the leaves infected with the Δ*ztvelB* mutant micropycnidiospore suspension only produced clear droplets as observed previously. As expected, the spore suspensions harvested from leaves infected with the IPO323 strain contained both micropycnidiospores and macropycnidiospores, however, the spore suspensions obtained from all three Δ*ztvelB* mutant strains only produced micropycnidiospores (Supplementary Figure S5). Taken together, these results demonstrate that the micropycnidiospores produced by the Δ*ztvelB* mutant pycnidia are viable and capable of causing infection on the wheat host.

## Discussion

Light is a key environmental signal which is perceived by fungi and used to regulate developmental processes. This cue can influence conserved processes, such as sporulation in both basidiomycete and ascomycete species, as well as species-specific adaptations including pathogenicity (Kim H. et al., 2011; Canessa et al., 2013; Fischer et al., 2016). For example, in the ascomycete plant pathogen *Cercospora zeae-maydis* (causal agent of grey leaf spot of maize), light is required for stomatal tropism and infection (Kim S. et al., 2011). However, despite the known importance of light in other fungal species, there is currently limited knowledge of the role of this signal for development in *Z. tritici.*

Previous studies have alluded to the potential role of light in *Z. tritici* development. For example, incubation of *Z. tritici* under either red, blue, or no light has been shown to affect growth of aerial mycelia (Choi and Goodwin, 2011). Our results show that white light supplemented with UV-A is detected by *Z. tritici*, and that this signal impacts vegetative growth. Light may therefore serve as an environmental cue which regulates key processes in *Z. tritici* such as morphology and pathogenicity potential, however, the genes involved in light detection and regulation of downstream processes in *Z. tritici* remain to be characterised in detail.

In *A. nidulans*, the light signal is detected by photoreceptor proteins which co-ordinate this signal with downstream processes, through the *velvet* genes *veA*, *velB*, *velC*, and *vosA* (Mooney and Yager, 1990; Ni and Yu, 2007; Park et al., 2012). Homologues to the *velvet* genes have been identified in both basidiomycete and ascomycete fungi, suggesting a conserved role in light signal processing. Until now, the only *velvet* gene identified in *Z. tritici* was *MVE-1* which is a homologue to *veA.* Deletion of this gene has been shown to impact development in *Z. tritici*, including aerial mycelia growth in response to light (Choi and Goodwin, 2011). In this study we identified the remaining three *velvet* genes in *Z. tritici*, *ZtvelB*, *ZtvelC*, and *ZtvosA.* Bioinformatic analyses highlighted a single homologue for each *A. nidulans* genes in *Z. tritici*, and all of these homologues contain the *velvet* domain.

We characterised a second *velvet* gene in *Z. tritici*, *ZtvelB*, and have shown that this gene is essential for normal vegetative development and formation of the asexual pycnidia and macropycnidiospores. Disruption of *ZtvelB* abolishes yeast-like growth *in vitro* and supports previous evidence that the *Z. tritici velvet* genes regulate vegetative growth in this pathogen. For example, deletion of the previously characterised *veA* homologue in *Z. tritici*, *MVE-1*, causes a significant reduction in the production of aerial mycelia. In addition, the Δ*mve-1* mutants do not respond to the light cues which trigger aerial mycelial growth in the wild-type (Choi and Goodwin, 2011). Our results show that the Δ*ztvelB* mutants have a hyphal-only phenotype under both LD and DD conditions, which suggests an inability to respond to the light cues tested in this study. In *A. nidulans*, the VelB protein is involved in co-ordinating the light response with downstream developmental and secondary metabolism pathways (Bayram et al., 2008). Therefore, evidence from the deletion of the *Z. tritici MVE-1* and *ZtvelB* genes suggests that the MVE-1 and ZtvelB proteins may be involved in co-ordinating upstream light signalling pathways with downstream processes such as hyphal development.

The essential role of the *velB* gene in vegetative growth is further supported by evidence from other fungal species. For example, deletion of the *C. lunata ClvelB* gene causes an increase in aerial hyphal growth (Gao et al., 2017). In contrast, deletion of the *F. graminearum FgvelB* suppresses aerial hyphal growth (Jiang et al., 2012). Therefore, the involvement of *velvet* genes in vegetative growth may be evolutionarily conserved among fungi, but the precise roles and morphological outcomes differ.

Previous data from the deletion of *velvet* homologues in other species has shown that these genes can be involved in pigmentation (Jiang et al., 2012; Park et al., 2012; Yang et al., 2013; Gao et al., 2017; Wu et al., 2018). The Δ*mve-1* and Δ*ztvelB* mutant phenotypes differ in that deletion of *MVE-1* abolishes melanisation, whilst deletion of *ZtvelB* does not. Therefore, the *Z. tritici velvet* genes have differing roles in melanisation in this fungus.

Despite the requirement of *ZtvelB* for yeast-like growth in *Z. tritici*, this morphology was not essential for successful infection of the host. Although timing of symptom appearance was delayed in the Δ*ztvelB* mutants, all three strains caused normal disease symptoms. We are cautious to conclude that the deletion of the *ZtvelB* gene delays infection as the results observed may be due to differences in the form of inoculum present in the IPO323 and Δ*ztvelB* cultures, rather than due to a slower disease progression in the mutants.

It is important to note that there is no documented evidence for the yeast-like phenotype being observed for *Z. tritici in planta*, therefore, the yeast-like growth form observed *in vitro* may not be essential for successful infection (Orton et al., 2011). Indeed, several previous observations have highlighted that, *in planta*, any yeast form of inoculum must transition to a hyphal form before the fungus can penetrate the stomata (Palmer and Skinner, 2002; Fones et al., 2017). This would explain why the Δ*ztvelB* mutants were still able to infect the host despite being unable to produce yeast-like cells.

Our results show that *ZtvelB* is not essential for initiation of pycnidia formation, as Δ*ztvelB* mutants were still able to form pycnidia-like structures *in vitro* or *in planta.* The number of pycnidia produced in the Δ*ztvelB* mutants was not consistently statistically significant and likely reflects the delayed disease progression of the Δ*ztvelB* mutants.

The ability to make pycnidia was not abolished in the Δ*ztvelB* mutants, but pycnidial structure differed from the IPO323 pycnidia. The Δ*ztvelB* mutant pycnidia were misshapen compared to the IPO323 pycnidia, they did not produce hyaline cirrhus and some of the pycnidia produced aerial hyphae from the surface or ostiole region. One possible explanation for the aerial hyphae observed in the Δ*ztvelB* mutant pycnidia is that the hyphal phenotype observed *in vitro* also affects the pycnidia development *in planta.* The aerial hyphae phenotype observed in this study was not documented for the Δ*mve-1* mutants, which only displayed abnormalities in pycnidia melanisation. Additionally, in other fungal species deletion of *velB* homologues either disrupts or increases asexual sporulation (Jiang et al., 2012; Kim et al., 2014; Wu et al., 2018). Therefore, to our knowledge, this is the first time that this fruiting body abnormity has been documented in *Z. tritici*.

In addition to the abnormal pycnidia structure, the Δ*ztvelB* mutants were severely disrupted in macropycnidiospore production and only produced micropycnidiospores. These micropycnidiospores were similar in size and structure to those previously recorded for *Z. tritici* (Sprague, 1950; Perelló et al., 1990; Eyal, 1999). No intermediate-sized cells were observed, which suggests that the phenotype documented is not due to delayed macropycnidiospore development.

Micropycnidiospores in *Z. tritici* were first observed in 1950, but it was not until 40 years later that these cells were demonstrated to act as a source of inoculum (Sprague, 1950; Perelló et al., 1990). Previous studies on asexual spores from *Z. tritici* demonstrated that field isolates can produce only macropycnidiospores, only micropycnidiospores or a combination of both spore types. Our results show that the IPO323 strain can produce both macropycnidiospores and micropycnidiospores, and that deletion of the *ZtvelB* gene only abolishes macropycnidiospore production, indicating a different regulatory pathway for each spore type.

Despite the impact on macropycnidiospore production, there was no statistical difference between the number of micropycnidospore produced between the IPO323 strain and Δ*ztvelB* mutants tested. However, it is important to note that micropycnidiospores can be difficult to distinguish from other cellular debris due to their small size. Additionally, the data obtained was highly zero inflated which may be due to clustering of the micropycnidiospores as observed in Figure 7C. Therefore, it is for this reason that we have been cautious to conclude whether there was significant difference between the number of micropycnidiospores produced by the IPO323 strain compared to the Δ*ztvelB* mutant strains.

Further analysis of the Δ*ztvelB* mutants showed that the micropycnidiospores harvested from the Δ*ztvelB* mutant strains were capable of causing infection similar to the IPO323 mixture of macropycnidiospores and micropycnidiospores. This agrees with previous evidence that micropycnidiospores can act as a source of inoculum during *Z. tritici* infection (Perelló et al., 1990).

The role of *ZtvelB* in asexual sporulation agrees with some findings from *velB* homologues in other fungal species. For example *MovelB* mutants of *M. oryzae* produce fewer conidia (Kim et al., 2014). In contrast, deletion mutants of the *V. mali VmvelB* gene and the *F. graminearum FgvelB* gene both have increased conidiation (Jiang et al., 2012; Wu et al., 2018). This suggests that the involvement of *velB* in asexual sporulation is conserved among fungi, but that the exact function of this gene can differ between species.

Our results suggest that the pathways for macropycnidiospore and micropycnidiospore formation in *Z. tritici* may be independently controlled. One theory we propose is that the genetic pathway controlling *Z. tritici* yeast-like growth also co-ordinates macropycnidiospore development. Micropycnidiospore production, on the other hand, may then be controlled through a separate set of genes such as those which regulate hyphal growth or sexual sporulation. It could be possible that micropycnidiospores serve a primary function as gametes for sexual sporulation with a secondary role as infection agents (Perelló et al., 1990). However, knowledge of sexual sporulation and the genes controlling this process in *Z. tritici* is severely limited. If the micropycnidiospores do act as the male gamete in sexual reproduction, then Δ*ztvelB* mutants would likely not be impaired in fertility.

Our findings add to the current understanding on the importance of light as an environmental signal in *Z. tritici*, and the role of *ZtvelB* as a co-ordinator for downstream developmental processes. There continues to be large gaps in our knowledge of the genetic components regulating key developmental process in *Z. tritici.* Therefore the Δ*ztvelB* mutants generated in this study serve as useful resources for future research, particularly focusing on the role of light, and the genetic basis of the dimorphic switch and sporulation in this species.

## Data Availability Statement

All datasets generated for this study are included in the manuscript/Supplementary Files.

## Author Contributions

AT designed and performed the experiments, analysed the data, and wrote the manuscript. HW carried out the statistics on the micropycnidiospore data and provided the feedback on the manuscript. AB devised the project, contributed to the experimental design and analysis, and provided the feedback on the manuscript. GF supervised the project and provided the feedback on the manuscript.

## Conflict of Interest

The authors declare that the research was conducted in the absence of any commercial or financial relationships that could be construed as a potential conflict of interest.

## Footnotes

1 http://genome.jgi-psf.org/Mycgr3/Mycgr3.home.html
