# Supplementary figures and images for: The ZtvelB Gene Is Required for Vegetative Growth and Sporulation in the Wheat Pathogen Zymoseptoria tritici
Source: Front Microbiol. 2019 Oct 1;10:2210. doi: 10.3389/fmicb.2019.02210 (PMC6779691; doi:10.3389/fmicb.2019.02210)

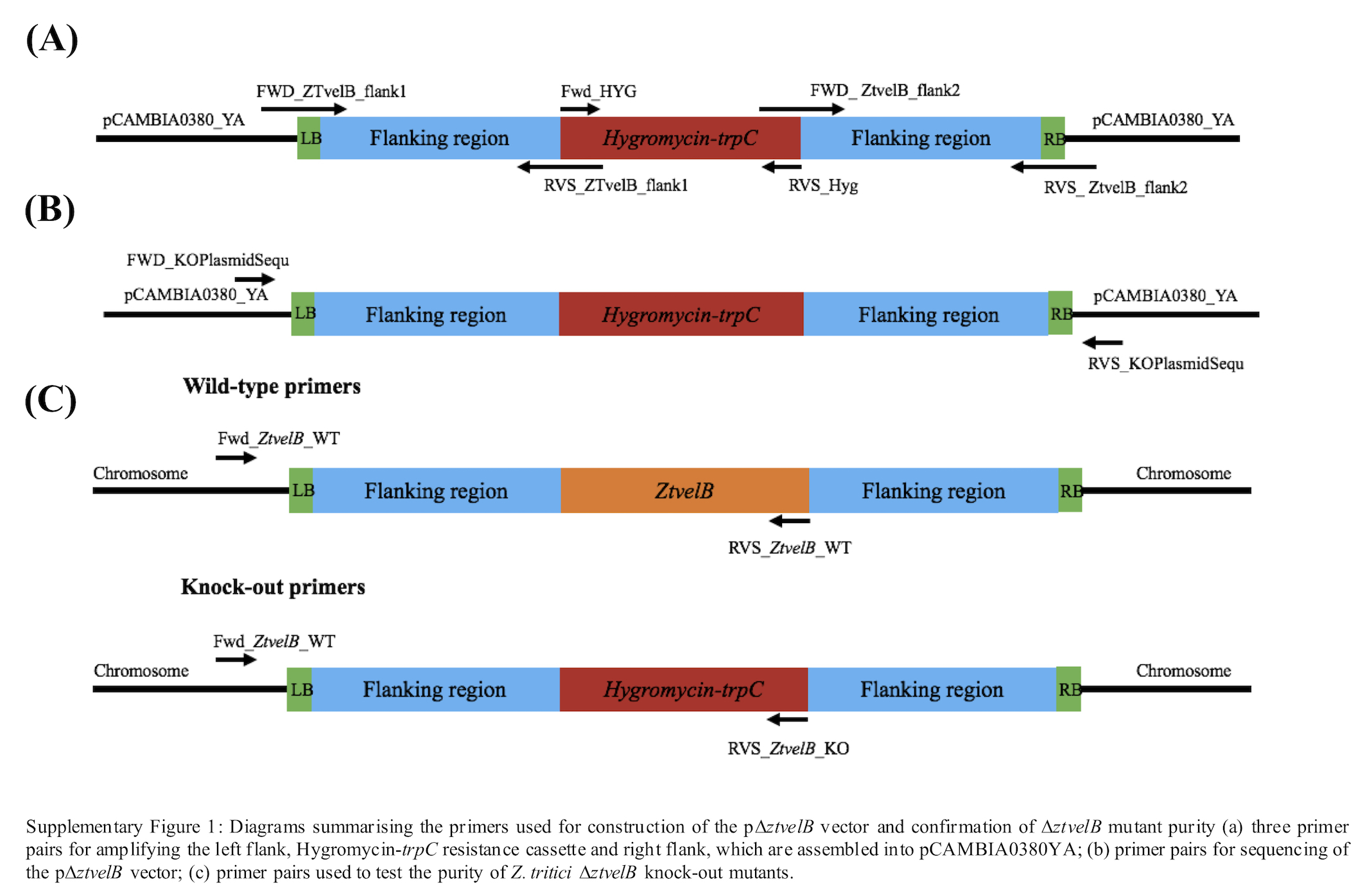

Supplement: Supplementary file 3 [file Image_1.JPEG]

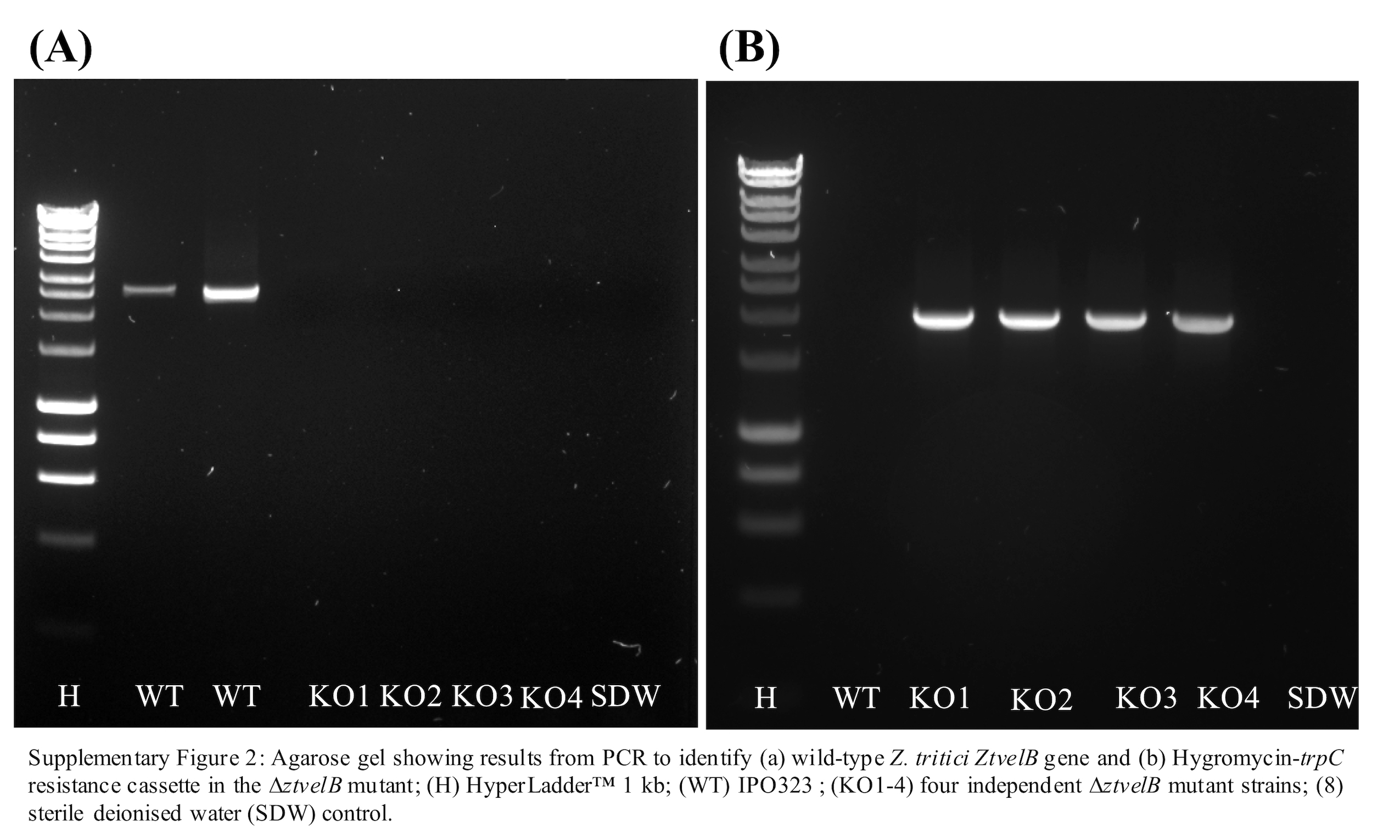

Supplement: Supplementary file 4 [file Image_2.JPEG]

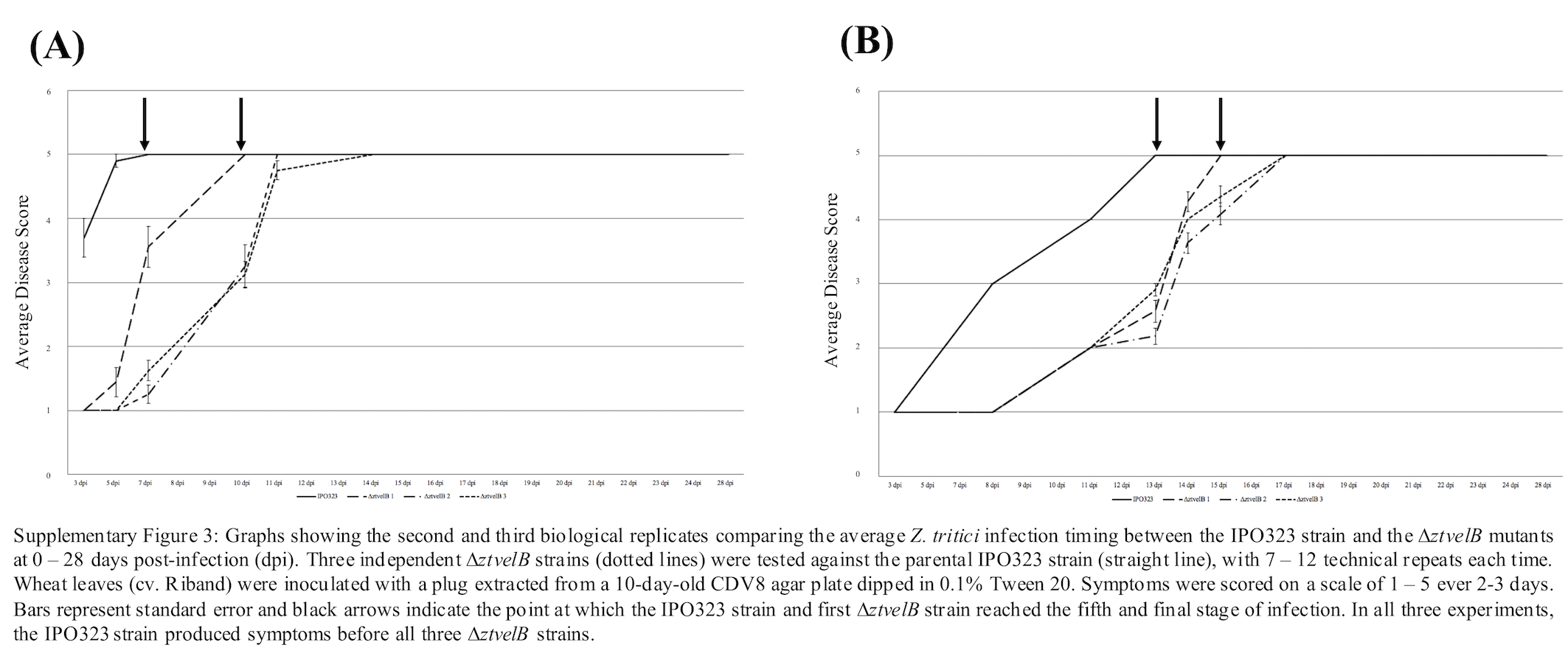

Supplement: Supplementary file 5 [file Image_3.JPEG]

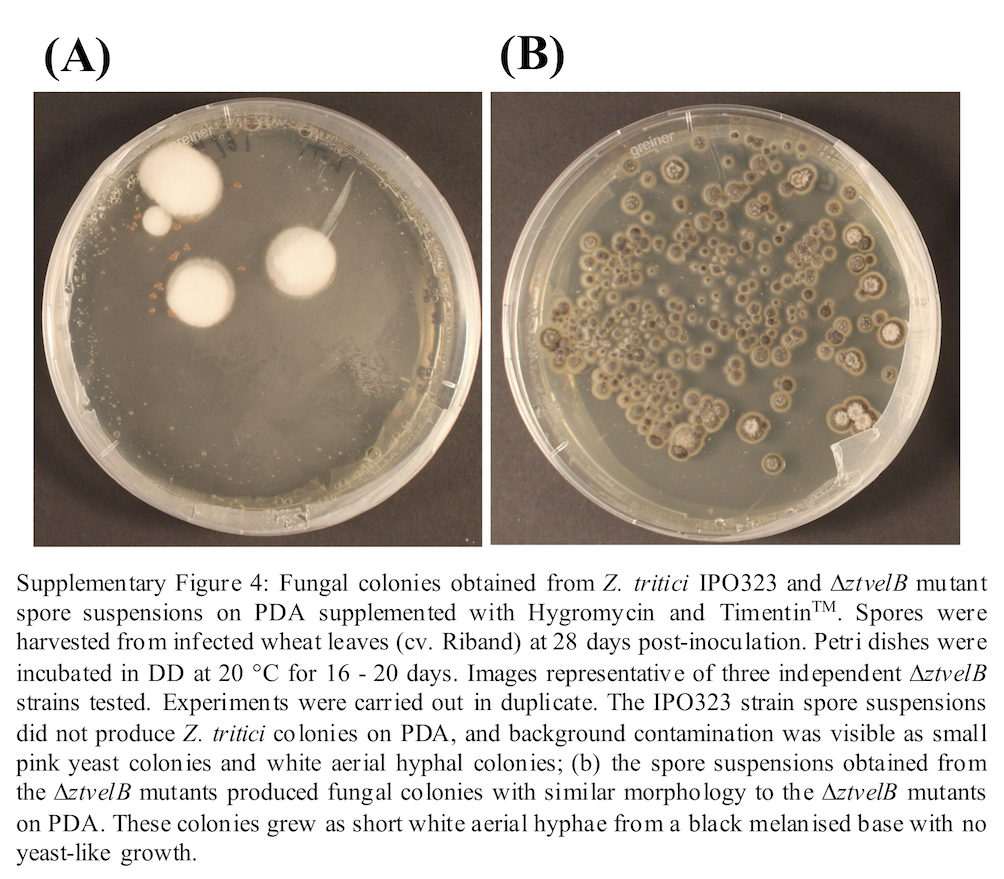

Supplement: Supplementary file 6 [file Image_4.JPEG]

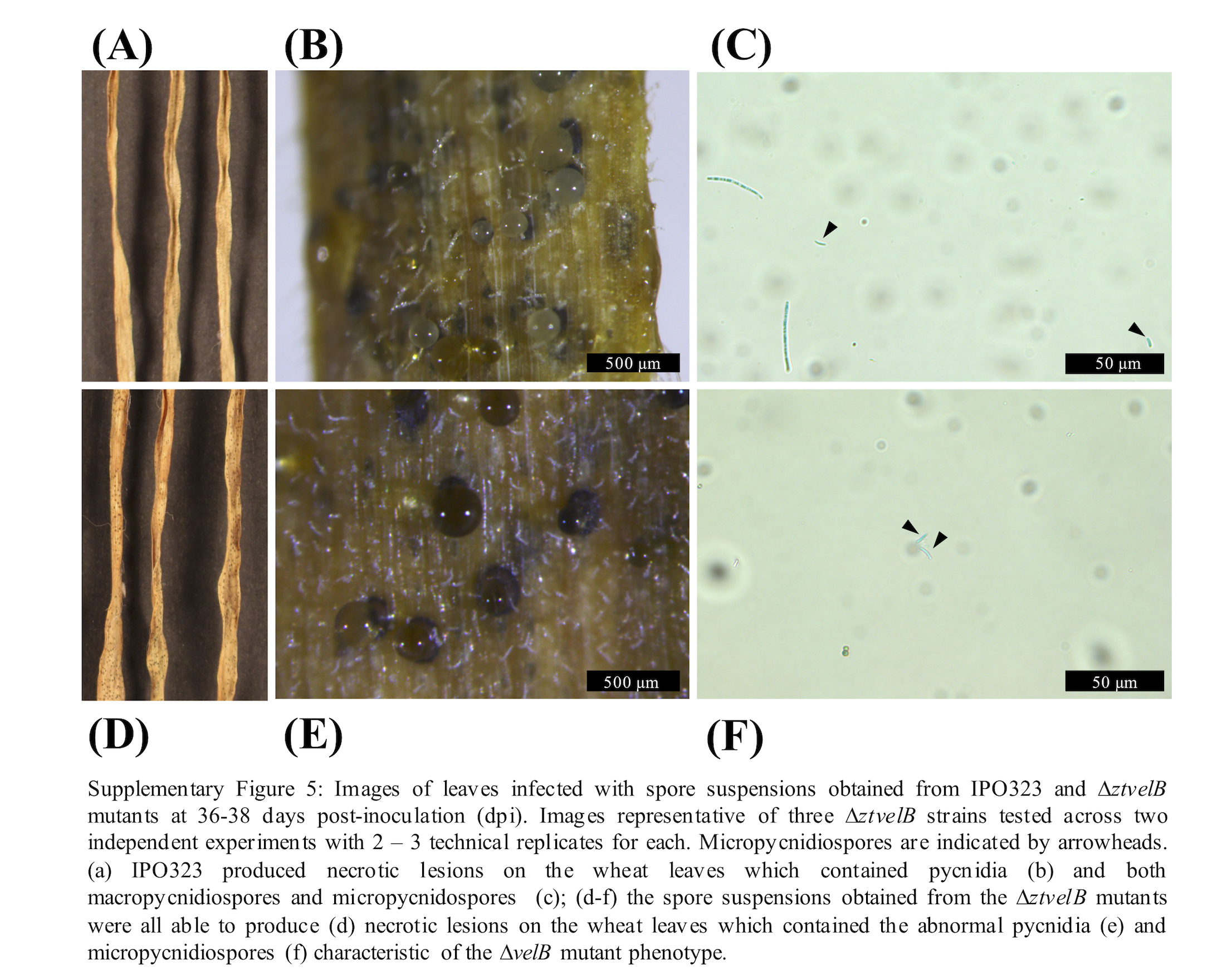

Supplement: Supplementary file 7 [file Image_5.JPEG]
